# Quality improvement initiative to optimize use of rapid genomic sequencing in a level IV NICU

**DOI:** 10.1038/s41372-025-02541-5

**Published:** 2026-01-12

**Authors:** Alissa M. D’Gama, Rachel S. Hu, Maya C. Del Rosario, Sonia Hills, Hannah J. Park, Anna-Thérèse Mehra, Laura S. Tannenbaum, Sarah U. Morton, Pankaj B. Agrawal, Monica H. Wojcik

**Affiliations:** 1 Division of Newborn Medicine, Department of Pediatrics, Boston Children’s Hospital, Boston, MA, USA.; 2 Harvard Medical School, Harvard University, Boston, MA, USA.; 3 Division of Genetics and Genomics, Department of Pediatrics, Boston Children’s Hospital, Boston, MA, USA.; 4 Present address: Bronson Healthcare, Kalamazoo, MI, USA.; 5 Present address: Center for Cardiovascular Genetics, Department of Cardiology, Boston Children’s Hospital, Boston, MA, USA.; 6 Present address: Division of Neonatology, Department of Pediatrics, University of Miami Miller School of Medicine and Holtz Children’s Hospital, Jackson Health System, Miami, FL, USA.

## Abstract

**OBJECTIVE:**

Optimize use of rapid genomic sequencing (rGS) in a level IV NICU.

**STUDY DESIGN:**

We designed interventions to improve patient identification, ordering processes, and provider education for rGS in our level IV NICU. We measured the percentage of infants eligible for rGS by internal criteria who had rGS sent, diagnostic yield of rGS (balancing measure), and days from genetics consult to rGS result (balancing measure).

**RESULT:**

Our study included 560 infants undergoing genetics evaluation. The percentage of eligible infants who had rGS sent significantly increased from 37% pre-intervention (January 2019–March 2021) to 54% post-intervention (April 2021–September 2024) (*p* < 0.001). Diagnostic yield of rGS remained stable (32% vs 34%). Time from genetics consult to rGS result significantly decreased from median 32 to 27 days (*p* = 0.04).

**CONCLUSION:**

Our quality improvement initiative increased rGS use with stable diagnostic yield and decreased time to rGS result for critically ill infants with suspected genetic disorders.

## INTRODUCTION

Many genetic disorders present in the perinatal period and contribute substantially to infant morbidity and mortality and healthcare system costs [1–3]. Infants with known or suspected genetic disorders often require specialized care after birth in Neonatal Intensive Care Units (NICUs), particularly in level IV NICUs that provide the highest level of care with access to medical and surgical subspecialists [4]. Early genetic testing during the NICU admission has the potential to identify genetic diagnoses, impact management, and improve outcomes for these infants [5]. For example, genetic testing results (both diagnostic and non-diagnostic) may have clinical and personal utility for the infant and their family, including informing treatment, workup, prognosis, and goals of care for the infant as well as reproductive planning and further workup for the infant’s family.

Diagnostic genomic sequencing (GS) tests, i.e., exome or genome sequencing, are comprehensive genome-wide approaches to identify many different underlying genetic diagnoses. Multiple studies have demonstrated that rapid genomic sequencing (rGS) tests have high utility for critically ill infants admitted to NICUs with suspected underlying genetic disorders [6]. However, access to these tests varies widely due to barriers including cost, expertise, and logistics [7]. A recent survey of 112 US NICUs demonstrated that >30% lack routine access to GS [8]. Furthermore, a study of 32 level IV NICUs found that access to GS was restricted in >80% and rGS was not available in >30% [9].

Prior studies have investigated rGS implementation in NICUs in the US and internationally, but limited data exist on quality improvement (QI) approaches to optimize rGS use after initial availability in this setting [10–14]. This is important as even NICUs with routine access to rGS have variable use of rGS and delays in the rGS process: we previously reported a pilot research study of rGS in our level IV NICU at Boston Children’s Hospital (BCH) [15] and the subsequent incorporation of rGS into routine clinical care in our NICU that identified areas for ongoing optimization, notably potential missed opportunities for rGS [16]. Our Neonatal Genomics Program (NGP) therefore launched a QI initiative in April 2021 with the goal of increasing the percentage of infants undergoing genetics evaluation in our NICU who were eligible for rGS and had rGS sent from 37 to 50% by September 2024.

## METHODS

### Context

The BCH NICU is an urban 30-bed level IV NICU in Boston, MA that cares for outborn infants. The rGS workflow prior to our QI initiative is described in detail in our previous study [16]. Briefly, the neonatology team consults the clinical genetics service to evaluate any admitted infant that the neonatology team suspects to have an underlying genetic disorder. Although a genetics consult is not required to order genetic testing, in practice the genetics team helps determine when genetic testing, including rGS, is indicated and facilitates applying for institutional approval for rGS and coordinating the process, including consent, ordering, sample collection, shipping to the off-site CLIA-certified vendor (GeneDx, Gaithersburg, MD), and results return [17].

### Interventions

Our multidisciplinary NGP combines expertise in neonatology and clinical genetics; the team initially included attending physicians, clinical fellows, neonatal nurse practitioners, neonatal nurses, and research assistants, and subsequently our inpatient genetic counselor (GC) and genetic counseling assistant (GCA). Beginning mid-summer 2020, members of our team began to systematically evaluate our approach to rGS in the NICU and to assist the team in test request and ordering as part of their clinical practice. This pre-intervention period informed our approaches once the official QI project was launched. We then undertook a QI initiative to increase appropriate use of rGS for infants in our NICU undergoing genetics evaluation through multiple interventions focused on patient identification, ordering optimization, and provider education. First, we helped identify clear criteria for rGS. Our eligibility criteria for rGS were: one or more congenital anomalies (with the exception of certain low-yield exclusion phenotypes), dysmorphic features, suspected metabolic disease, neurologic features, family history of genetic disorder without molecular genetic diagnosis in family members, failure to thrive, and/or high suspicion for another Mendelian disorder (e.g., genetic surfactant deficiency). Our exclusion criteria were: previous GS, historically low yield phenotype (non-syndromic VACTERL, OEIS, or isolated esophageal atresia/tracheoesophageal fistula, omphalocele, or gastroschisis), family history of a genetic disorder with known molecular genetic diagnosis in family members that matches the infant’s phenotype (where targeted genetic testing may be more appropriate), features strongly suggestive of specific chromosomal anomaly (e.g., Trisomy 21, where targeted genetic testing may be more appropriate), and/or clear non-genetic explanation for the infant’s presentation (e.g., early-onset sepsis). We performed biweekly reviews of NICU genetics consultations to identify infants who qualified for rGS. We then followed up with on-service clinical neonatology and genetics teams for infants who met phenotypic criteria but did not have rGS sent (started April 2021 and ongoing) to advocate for use of rGS. Second, we educated NICU providers on criteria for rGS at a neonatal nurse practitioner retreat and developed a handout for easy reference (March 2022). Third, we worked with our Laboratory Medicine department to simplify the rGS institutional approval form and developed a step-by-step rGS ordering workflow handout for NICU staff (April-May 2022). Fourth, we re-educated NICU staff after our NICU physically moved and expanded from 24 to 30 beds. Fifth, an inpatient GCA joined the genetics team and began attending NGP meetings (started July 2023) to facilitate discussion between the on-service clinical neonatology and genetics teams, and sixth, an inpatient GC similarly joined these team meetings (started July 2024).

### Study of the interventions

We collected pre-intervention data from January 2019 to March 2021 and post-intervention data from April 2021 to September 2024 for infants admitted to our level IV NICU undergoing genetics evaluation. Infants with a previously known molecular genetic diagnosis who had genetics consults to provide further counseling to the family or assist the neonatology team in further management were not included. Demographic, clinical, and genetic testing data were abstracted from the electronic medical record (EMR) and stored in a BCH-hosted Research Electronic Data Capture database [18].

### Measures

Our main measure was the percentage of NICU infants undergoing genetics evaluation who met criteria for rGS and had rGS sent. To assess if increased frequency of rGS may lead to potentially inappropriate use of rGS for infants with low likelihood of an underlying genetic disorder, we measured the diagnostic yield of rGS sent. An rGS result was considered diagnostic if pathogenic or likely pathogenic variant(s) by ACMG criteria [19] were reported that explained the infant’s phenotype or variants of uncertain significance (VUS) were reported that the clinical team deemed clinically diagnostic based on clinical features or further investigations. To assess if increased use of rGS may lead to additional delays in the rGS process, we measured the time from genetics consult to rGS result (days), and the times from genetics consult to sample collection and from sample collection to rGS result (days).

### Analysis

Demographic and clinical characteristics and the above measures were compared between the pre- and post-intervention cohorts using chi-square, Fisher’s exact, or Mann–Whitney U tests as appropriate using SPSS. Statistical process charts (p and Xbar) were created and analyzed in Excel using QI Macros to identify special cause variation for the above measures.

## RESULTS

### Cohort

Our study included 560 infants who underwent genetics evaluation in our level IV NICU: 224 in the pre-intervention cohort and 336 in the post-intervention cohort. There was no significant difference in sex, gestational age, birth weight, or age at genetics consult between the cohorts (Table 1).

### Use of rGS

**Fig. 1:**
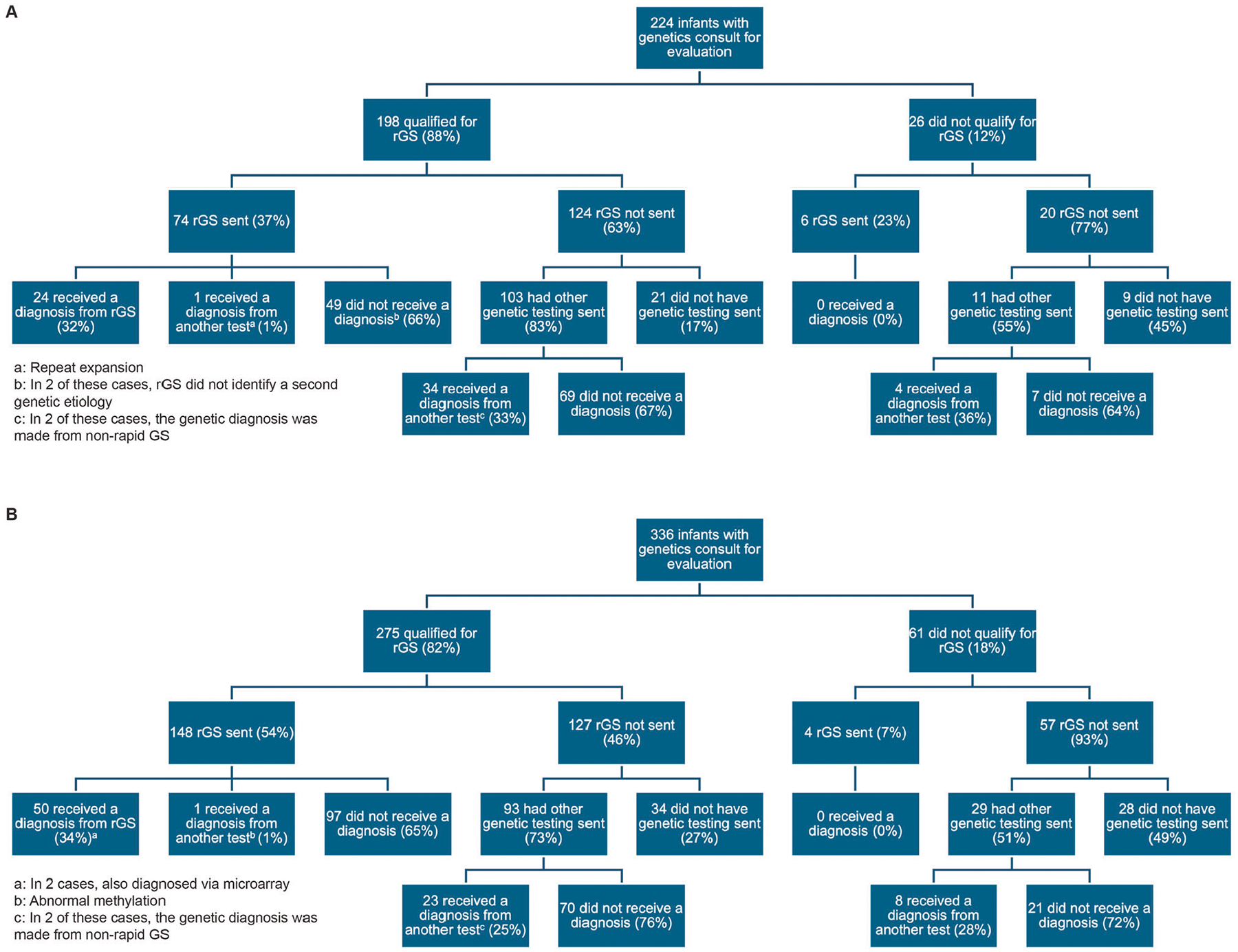
Rapid genomic sequencing in the NICU. Flowchart of the infants analyzed in the **A** pre-intervention and **B** post-intervention phases, including genetic testing and genetic diagnoses.

**Fig. 2:**
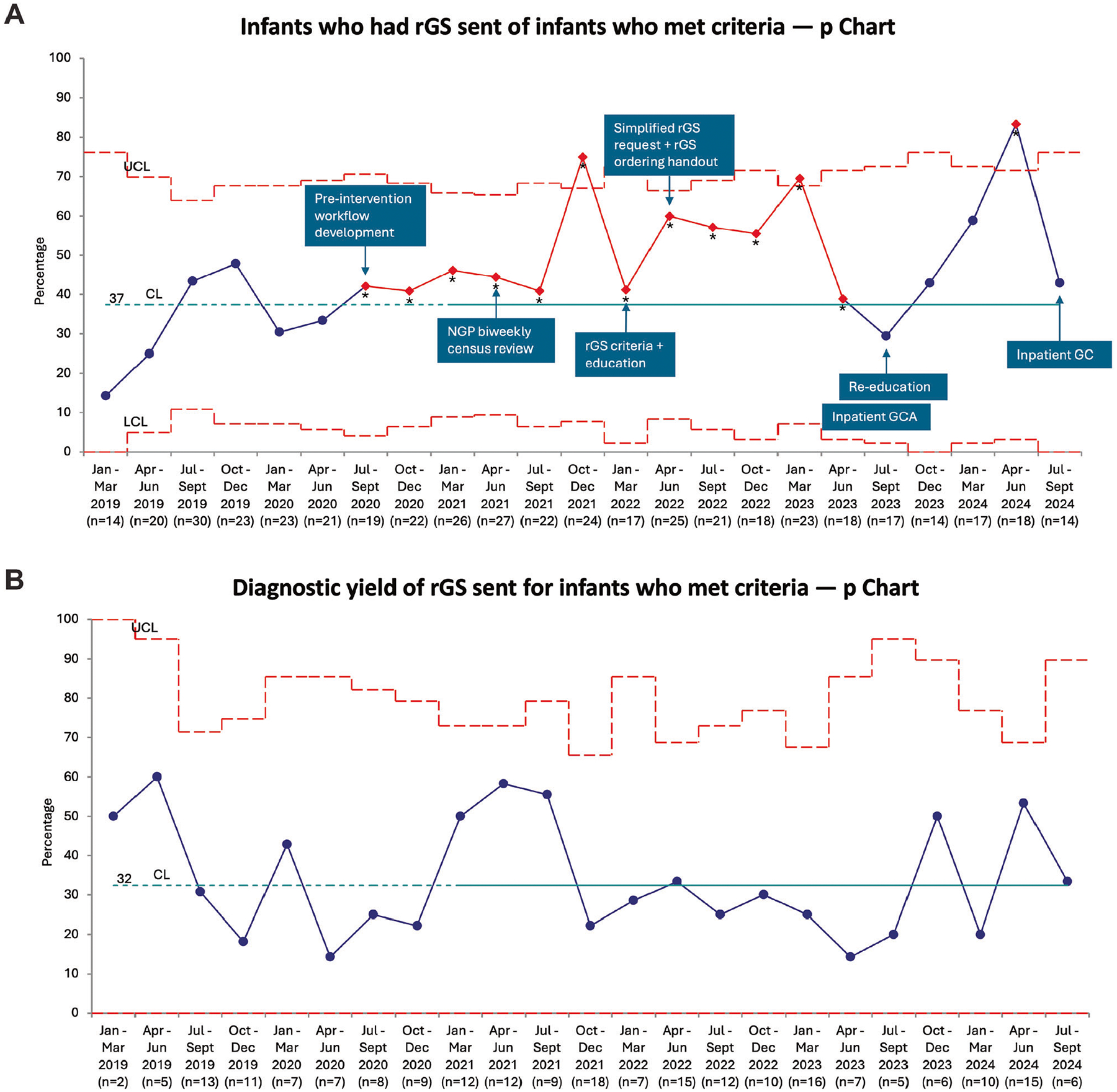
Use and yield of rGS. **A** A p chart displaying the percentage of infants eligible for rGS who had rGS sent with annotated interventions. **B** A p chart displaying the diagnostic yield of rGS sent for eligible infants. The CL was calculated based on the pre-intervention data (dotted portion of the line). Special cause variation is depicted in red and with as asterisk. CL centerline, LCL lower control limit, UCL upper control limit.

Using our criteria, 88% (198/224) of infants in the pre-intervention cohort and 82% (275/336) in the post-intervention cohort qualified for rGS (Fig. 1A, B). The most common inclusion criteria were one or more congenital anomalies and dysmorphic features, while the most common exclusion criteria were previous GS and low-yield phenotypes (Table 1). The percentage of infants who qualified for rGS and had rGS sent significantly increased from 37% (74/198) in the pre-intervention cohort to 54% (148/275) in the post-intervention cohort (*p* < 0.001), with special cause variation noted on the *p* chart (Table 2, Fig. 2A). Our pre-intervention trials of support for test request and ordering resulted in improvement in testing sent even before the official launch of our QI project (Fig. 2A).

Of the 124 infants in the pre-intervention cohort who qualified but did not have rGS sent, 103 (83%) had other genetic testing sent and 21 (17%) did not have any genetic testing sent, including 4 where parents declined genetic testing. Of the 127 infants in the post-intervention cohort who qualified but did not have rGS sent, 93 (73%) had other genetic testing sent and 34 (27%) did not have any genetic testing sent, including 8 where parents declined genetic testing. In addition, we observed that the time from genetics consult to NICU discharge was higher in the cohort who had rGS sent compared to those who did not (median time from consult to discharge 16 days [IQR 5–37] for infants who were eligible and did not have rGS sent vs 29 days [IQR 15–86] for those who did, *p* < 0.001).

### Yield of rGS

The diagnostic yield of rGS sent for infants who qualified did not significantly differ between the pre-intervention (32% [24/74]) and post-intervention (34% [50/148]) cohorts (*p* = 0.84; Table 2, Fig. 2B, Supplementary Table 1). One infant in each cohort had non-diagnostic rGS and received a genetic diagnosis from another test (repeat expansion and abnormal methylation signature; both not detectable by rGS technology when the test was sent). Of the infants who qualified but did not have rGS sent and had other genetic testing sent, 33% (34/103) in the pre-intervention cohort and 25% (23/93) in the post-intervention cohort received genetic diagnoses, including 2 infants in each cohort who received diagnoses from non-rapid GS. For infants who did not qualify but still had rGS sent by the clinical team (5 with low-yield phenotypes, 3 with previous GS, 1 with cord blood testing, 1 with non-genetic explanation), the yield of rGS was 0% in both pre-intervention (0/6) and post-intervention (0/4) cohorts (Fig. 1).

### Turnaround time of rGS

**Fig. 3:**
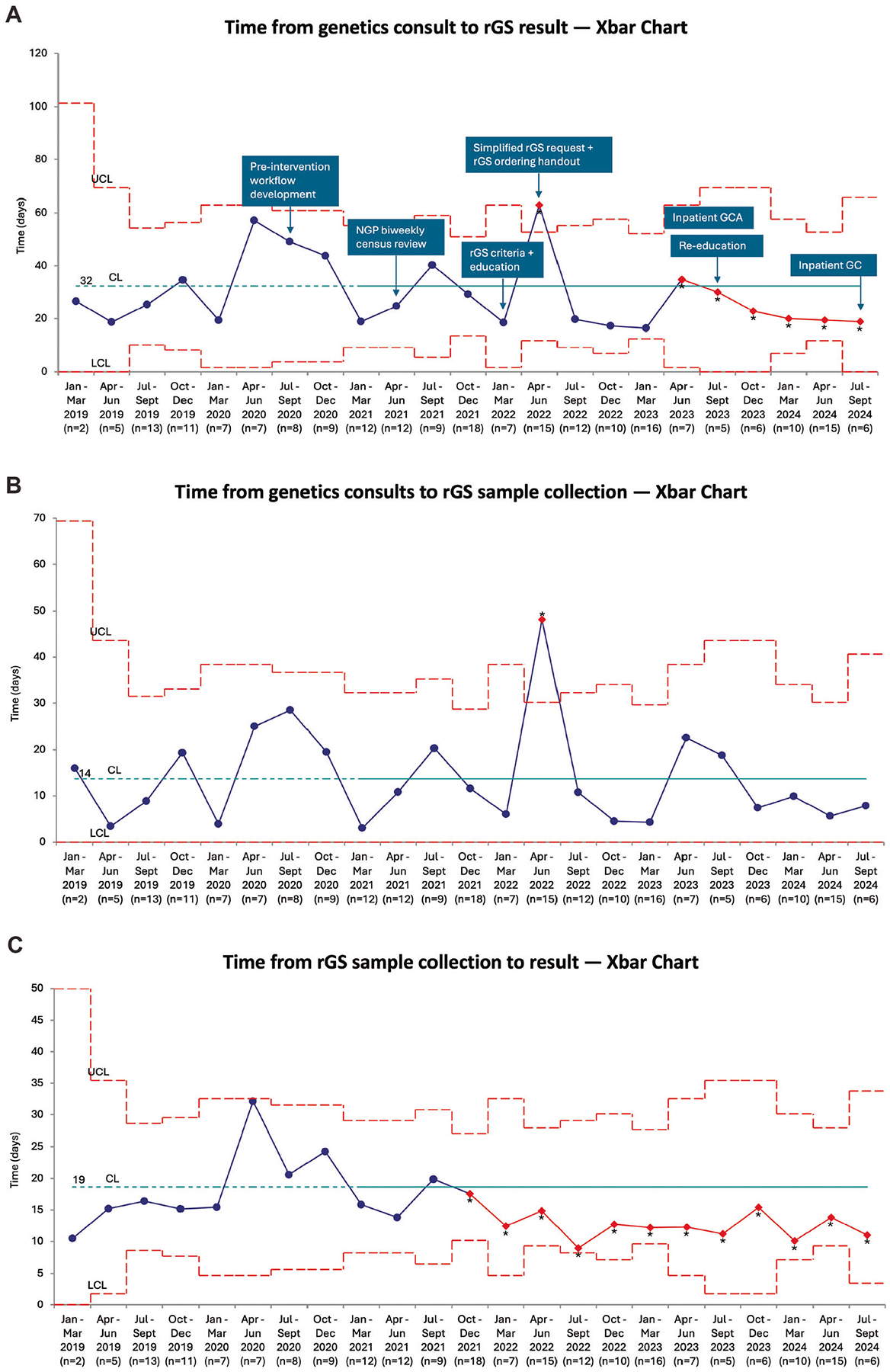
Turnaround time of rGS. Xbar charts displaying the average time (days) from **A** genetics consult to rGS result with annotated interventions, **B** genetics consult to sample collection and **C** sample collection to rGS result. The CL was calculated based on the pre-intervention data (dotted portion of the line). Special cause variation is depicted in red and with as asterisk. CL centerline, LCL lower control limit, UCL upper control limit.

The turnaround time (days from genetics consult to rGS result) significantly decreased from median 20 days (interquartile range [IQR] 16–27) in the pre-intervention cohort to median 18 days (IQR 13–26) in the post-intervention cohort (*p* = 0.04), with special cause variation noted on the Xbar chart (Table 2, Fig. 3A). The time from genetics consult to sample collection was not significantly different between the cohorts (Table 2, Fig. 3B). The time from sample collection to rGS result significantly decreased from median 14 days (IQR 12–19) in the pre-intervention cohort to median 12 days (IQR 8–15) in the post-intervention cohort (*p* < 0.001), with special cause variation noted on the Xbar chart (Table 2, Fig. 3C).

## DISCUSSION

### Summary

In this QI initiative, through multiple interventions, including enhanced patient identification using standardized criteria with ongoing review of NICU genetics consults, optimization of a standardized rGS workflow, and provider education, we increased appropriate use of rGS as reflected by increased use of rGS with stable diagnostic yield. Turnaround time to return of rGS results also decreased.

### Interpretation

We aimed to increase the percentage of infants undergoing genetics evaluation in our NICU who met criteria for rGS and had rGS sent from 37% to 50%. We achieved this goal with an increase to 54% in the post-intervention cohort, although p chart analysis demonstrated that the percentage varied quarter to quarter. Regular provider education of standardized criteria and workflow seems to have had the biggest impact on maintaining increased rGS use, with increases noted after both the March 2022 and July 2023 education interventions. The decrease April-Sept 2023 may reflect waning provider education after the initial education intervention, and the recent decrease July-Sept 2024 may again reflect waning provider education coupled with our transition to a new EMR system in June 2024.

As there are no current consensus criteria for rGS in NICUs, we developed phenotypic criteria for our unit based on literature review, expert opinion, and previous studies of rGS in our unit. Our QI inclusion criteria were broader than those in our pilot research study, as our analysis of the initial implementation of rGS into routine clinic care in our unit found that the relatively strict inclusion criteria of the pilot research study would have missed several diagnoses made during the initial implementation period (during which the decision to use rGS was made by individual clinical providers) [15, 16]. We also added exclusion criteria for 1) cases where rGS is currently known to be low yield (e.g., VACTERL association) and 2) cases where sending a different genetic test would be clinically appropriate, not delay genetic diagnosis, and be cost-effective (e.g., sending a karyotype instead of rGS for an infant with clinical features strongly suggestive of Trisomy 21). We were encouraged to find that very few infants who did not meet our criteria had rGS sent by the clinical team and none of those infants received a genetic diagnosis from rGS, suggesting our criteria were reasonable. Importantly, phenotype alone does not dictate access to testing in our NICU, because even infants who met phenotype criteria may not have rGS sent if anticipated to be discharged home prior to return of rGS results. As discharge or transfer date may be difficult to predict in a busy level IV NICU, we set our goal at 50% to accommodate this balancing factor. The shorter NICU length of stay that we identified for infants who did not have rGS sent compared to those who did likely reflects this practice pattern and provides further justification of our goal.

One of our balancing measures was the diagnostic yield of rGS sent, as we wanted to ensure that we increased appropriate use of rGS (for infants with reasonable likelihood of an underlying genetic diagnosis) rather than merely increasing usage and including infants who may be less likely to benefit. We demonstrated increased rGS use with stable diagnostic yield, which we interpret as appropriate increased use resulting in more infants receiving a genetic diagnosis from rGS in the post-intervention cohort. Our yield is similar to the average yield reported in a recent review of rGS in NICU settings [6]. This suggests that there were some infants in our pre-intervention cohort, and likely also our post-intervention cohort, who qualified for rGS but did not have rGS sent and thus have delayed or missed genetic diagnoses. Indeed, two infants in each cohort who qualified for rGS but did not have rGS sent subsequently received delayed genetic diagnoses by non-rapid GS. Although not one of our main measures, it was encouraging that only two diagnoses across the cohorts were “missed” by rGS—in both cases because the variant types were not technically possible to detect by rGS—highlighting the utility of rGS as a single test that can detect the vast majority of pathogenic variants.

Our other balancing measure was the turnaround time of rGS, defined as the time from genetics consult to rGS result, as we wanted to ensure that increased use of rGS did not introduce additional delays in the rGS process. The turnaround time includes two intervals: time from genetics consult to (proband) sample collection and time from sample collection to rGS result. The former encompasses the initial genetics evaluation, decision to use rGS, request for and institutional approval to order rGS, consenting the family, placing the rGS order, and collecting samples. The latter encompasses shipping the proband (and if applicable parental) samples to the rGS vendor and the sequencing, analysis, and rGS report performed by the vendor. While we demonstrated a decrease in turnaround time, review of the Xbar charts suggests that the decrease in the second time interval largely accounted for the overall decrease. Thus, the decrease appears largely due to the rGS vendor, who guaranteed the report within 14 days of sample arrival, being able to deliver reports faster over our post-intervention period.

Previous QI studies of rGS implementation in US NICU settings (e.g., Project Baby Bear in California, Project Baby Deer in Michigan, Project Baby Manatee in Florida) and internationally (e.g., Victorian Clinical Genetics Service in Australia) have highlighted the importance of “clinical champions” and standardized approaches [10, 11, 14]. In our study, which focused on increasing use of rGS after implementation, we similarly developed a multidisciplinary team of clinical champions (the NGP) and our interventions included standardized approaches for criteria and workflow, with provider education for awareness of these standardized approaches. Our study highlights the importance of ongoing QI initiatives after rGS implementation in NICU settings to increase appropriate use of rGS and shorten the diagnostic odyssey for critically ill infants with underlying genetic disorders. Moreover, our ability to identify and implement phenotypic criteria for rGS reflects the ability of our clinical and NGP teams to phenotypically assess NICU infants and the importance of ongoing communication between neonatology and genetics teams to determine the likelihood of Mendelian disease in a particular case. For example, we do not perform rGS for every case of hypoxic-ischemic encephalopathy because our teams have been quite accurate at predicting which infants have straightforward HIE and which have rare genetic disorders masquerading as perinatal complications.

### Limitations

Our study has several limitations. First, the increase in rGS use to 54% of eligible infants means that 46% still did not have rGS sent. In some of these cases, the clinical team may have wanted to send rGS but had their request denied by the institutional committee or may have been determined by the NICU or genetics teams to be close enough to hospital discharge that rGS was not indicated; we are not able to determine these outcomes via EMR review. Ultimately, we hope to continue to narrow this gap with future PDSA cycles, including tracking institutional requests. Second, the decrease in rGS turnaround time was largely driven by a decrease in vendor sequencing and analysis time. We hope to decrease time for the steps in our control through future PDSA cycles, including the recent addition of an inpatient GC who may help reduce delays in consenting and obtaining parental samples. Third, consensus criteria for rGS in NICU settings do not exist and the true diagnostic yield of GS in infants admitted to level IV NICUs is unknown. Thus, our criteria were developed by our clinical champions for use in our unit and represent a single-center experience. Finally, in a small but important number of cases, parents declined genetic testing including rGS, and this is not always accurately captured in our EMR. However, it is critical to understand parental perceptions to continue to optimize rGS use in this vulnerable population.

## CONCLUSION

Proactive identification of appropriate infants using standardized criteria, optimization of a standardized workflow, and education of NICU staff on these standardized approaches can lead to an increase in appropriate use of rGS in the NICU. Future studies should develop consensus criteria for rGS on a multi-site or national level to facilitate broader use of rGS for critically ill infants.

## Supplementary Material

Supplementary Material

**Supplementary information** The online version contains supplementary material available at https://doi.org/10.1038/s41372-025-02541-5.

## Figures and Tables

**Table 1.** Demographic and clinical characteristics.

(*N* (%) unless otherwise noted)	Pre-intervention (Jan 2019-Mar 2021, *n* = 224)	Post-intervention (Apr 2021-Sept 2024, *n* = 336)	*p* value
Male sex	129 (58)	203 (60)	0.51
Gestational age (weeks; median (IQR))	37 (34, 39)	37 (34, 39)	0.79
Birth weight (grams; median (IQR))	2670 (1990, 3200)	2595 (1960, 3199)	0.77
Age at genetics consult (days; median (IQR))	9 (3, 36)	7 (3, 33)	0.18
**Qualified for rGS**	198 (88)	275 (82)	0.04
*Inclusion criteria* ^a^			
One or more congenital anomalies	122 (62)	149 (54)	0.11
Suspected metabolic disease	50 (25)	59 (21)	0.33
Neurologic (seizures, hypotonia, etc.)	44 (22)	56 (20)	0.63
Family history of a genetic disorder (without known molecular genetic diagnosis in family members)	6 (3)	9 (3)	0.88
Dysmorphic features	91 (46)	86 (31)	0.001
Failure to thrive	6 (3)	8 (3)	0.94
Other suspected Mendelian disorder	55 (28)	87 (32)	0.37
**Did not qualify for rGS**	26 (12)	61 (18)	–
*Exclusion criteria* ^a^			
Previous GS	8 (31)	25 (41)	0.37
Low yield phenotype (VACTERL, OEIS, or isolated EA/TEF, omphalocele, or gastroschisis)	10 (38)	16 (26)	0.25
Family history of genetic disorder with known molecular genetic diagnosis in family members that matches infant phenotype	2 (8)	6 (10)	1
Features strongly suggestive of specific chromosomal anomaly (e.g., Trisomy 21)	1 (4)	4 (7)	1
Other (e.g., clear non-genetic explanation)	5 (19)	12 (20)	0.96

a Infants could meet more than one inclusion or exclusion criteria.

**Table 2.** Summary of measures.

	Qualified for rGS pre-intervention (*n* = 198)	Qualified for rGS post-intervention (*n* = 275)	*p* value
rGS sent (number (%))	74 (37)	148 (54)	<0.001
Diagnostic yield of rGS sent (number (%))	24 (32)	50 (34)	0.84
Interval from genetics consult to sample collection (days; median (IQR))	4 (2, 8)	4 (2, 10)	0.71
Interval from sample collection to rGS report (days; median (IQR))	14 (12, 19)	12 (8, 15)	<0.001
Interval from genetics consult to rGS report (days; median (IQR))	20 (16, 27)	18 (13, 26)	0.04

## References

[1] Almli LM, Ely DM, Ailes EC, Abouk R, Grosse SD, Isenburg JL, Infant mortality attributable to birth defects - United States, 2003–2017. MMWR Morb Mortal Wkly Rep. 2020;69:25–9. 31945037 10.15585/mmwr.mm6902a1 PMC6973351

[2] Heron M Deaths: leading causes for 2019. Natl Vital Stat Rep. 2021;70:1–114. 34520342

[3] Saunders CJ, Miller NA, Soden SE, Dinwiddie DL, Noll A, Alnadi NA, Rapid whole-genome sequencing for genetic disease diagnosis in neonatal intensive care units. Sci Transl Med. 2012;4:154ra135. 10.1126/scitranslmed.3004041 PMC4283791 23035047

[4] Stark AR, Pursley DM, Papile LA, Eichenwald EC, Hankins CT, Buck RK, Standards for levels of neonatal care: II, III, and IV. Pediatrics. 2023;151:e2023061957. 37212022 10.1542/peds.2023-061957

[5] Group NIS, Krantz ID, Medne L, Weatherly JM, Wild KT, Biswas S, Effect of whole-genome sequencing on the clinical management of acutely ill infants with suspected genetic disease: a randomized clinical trial. JAMA Pediatr. 2021;175:1218–26. 34570182 10.1001/jamapediatrics.2021.3496 PMC8477301

[6] Kingsmore SF, Cole FS. The role of genome sequencing in neonatal intensive care units. Annu Rev Genom Hum Genet. 2022;23:427–48. 10.1146/annurev-genom-120921-103442 PMC9844117 35676073

[7] D’Gama AM, Wojcik MH, Hills S, Douglas J, Network V, Yu TW, It’s hard to wait”: Provider perspectives on current genomic care in safety-net NICUs. Genet Med. 2024;26:101177. 38855852 10.1016/j.gim.2024.101177 PMC11380591

[8] Wojcik MH, Del Rosario MC, Agrawal PB. Perspectives of United States neonatologists on genetic testing practices. Genet Med. 2022;24:1372–7. 35304021 10.1016/j.gim.2022.02.009 PMC9272826

[9] Wojcik MH, Callahan KP, Antoniou A, Del Rosario MC, Brunelli L, ElHassan NO, Provision and availability of genomic medicine services in Level IV neonatal intensive care units. Genet Med. 2023;25:100926. 37422715 10.1016/j.gim.2023.100926 PMC10592224

[10] Best S, Brown H, Lunke S, Patel C, Pinner J, Barnett CP, Learning from scaling up ultra-rapid genomic testing for critically ill children to a national level. NPJ Genom Med. 2021;6:5. 33510162 10.1038/s41525-020-00168-3 PMC7843635

[11] Bupp CP, Ames EG, Arenchild MK, Caylor S, Dimmock DP, Fakhoury JD, Breaking barriers to rapid whole genome sequencing in pediatrics: Michigan’s Project Baby Deer. Children. 2023;10:106. 36670656 10.3390/children10010106 PMC9857227

[12] Diaby V, Babcock A, Huang Y, Moussa RK, Espinal PS, Janvier M, Real-world economic evaluation of prospective rapid whole-genome sequencing compared to a matched retrospective cohort of critically ill pediatric patients in the United States. Pharmacogenomics J. 2022;22:223–9. 35436997 10.1038/s41397-022-00277-5

[13] Dimmock D, Caylor S, Waldman B, Benson W, Ashburner C, Carmichael JL, Project Baby Bear: rapid precision care incorporating rWGS in 5 California children’s hospitals demonstrates improved clinical outcomes and reduced costs of care. Am J Hum Genet. 2021;108:1231–8. 34089648 10.1016/j.ajhg.2021.05.008 PMC8322922

[14] Franck LS, Kriz RM, Rego S, Garman K, Hobbs C, Dimmock D. Implementing rapid whole-genome sequencing in critical care: a qualitative study of facilitators and barriers to new technology adoption. J Pediatr. 2021;237:237–243.e232. 34023348 10.1016/j.jpeds.2021.05.045

[15] Gubbels CS, VanNoy GE, Madden JA, Copenheaver D, Yang S, Wojcik MH, Prospective, phenotype-driven selection of critically ill neonates for rapid exome sequencing is associated with high diagnostic yield. Genet Med. 2020;22:736–44. 31780822 10.1038/s41436-019-0708-6 PMC7127968

[16] D’Gama AM, Del Rosario MC, Bresnahan MA, Yu TW, Wojcik MH, Agrawal PB. Integrating rapid exome sequencing into NICU clinical care after a pilot research study. NPJ Genom Med. 2022;7:51. 36064943 10.1038/s41525-022-00326-9 PMC9441819

[17] Retterer K, Juusola J, Cho MT, Vitazka P, Millan F, Gibellini F, Clinical application of whole-exome sequencing across clinical indications. Genet Med. 2016;18:696–704. 26633542 10.1038/gim.2015.148

[18] Harris PA, Taylor R, Thielke R, Payne J, Gonzalez N, Conde JG. Research electronic data capture (REDCap)–a metadata-driven methodology and workflow process for providing translational research informatics support. J Biomed Inform. 2009;42:377–81. 18929686 10.1016/j.jbi.2008.08.010 PMC2700030

[19] Richards S, Aziz N, Bale S, Bick D, Das S, Gastier-Foster J, Standards and guidelines for the interpretation of sequence variants: a joint consensus recommendation of the American College of Medical Genetics and Genomics and the Association for Molecular Pathology. Genet Med. 2015;17:405–24. 25741868 10.1038/gim.2015.30 PMC4544753

[20] Ogrinc G, Davies L, Goodman D, Batalden P, Davidoff F, Stevens D. SQUIRE 2.0 (Standards for QUality Improvement Reporting Excellence): revised publication guidelines from a detailed consensus process. Am J Med Qual. 2015;30:543–9. 26497490 10.1177/1062860615605176 PMC4620592

