# Supplementary material for: Quality improvement initiative to optimize use of rapid genomic sequencing in a level IV NICU
Source: J Perinatol. Author manuscript; Available in PMC 2026 Mar 2. (PMC12952907; doi:10.1038/s41372-025-02541-5)
Supplement: Supplementary Material [file NIHMS2148640-supplement-Supplementary_Material.pdf]

Supplementary Table 1: Diagnoses from rGS

Gene/Genomic Region	Diagnosis
**Pre-intervention cohort**	
**4q34.3q35.2, 8q21.12q24.3**	4q34.3q35.2 deletion, 8q21.12q24.3 duplication
**ADNP**	Helsmoortel-van der Aa syndrome
**AIRE**	Autoimmune polyendocrinopathy syndrome, type I
**ALG12**	Congenital disorder of glycosylation, type Ig
**CA5A**	Carbonic anhydrase VA deficiency
**COL11A2**	Otospondylomegaepiphyseal Dysplasia
**DGAT1**	Diarrhea 7, protein-losing enteropathy type
**DHCR7**	Smith-Lemli-Opitz Syndrome
**HPRT1**	Lesch-Nyhan syndrome
**KMT2D**	Kabuki syndrome 1
**MMP21**	Heterotaxy, visceral, 7, autosomal
**MUSK**	Congenital myasthenic syndrome 9, associated with acetylcholine receptor deficiency
**NOTCH1**	Adams-Oliver syndrome 5
**P4HTM**	H ypotonia, hypoventilation, impaired intellectual development, dysautonomia, epilepsy, and eye abnormalities
**PEX1**	Peroxisome biogenesis disorder 1A (Zellweger syndrome)
**PIGV**	Hyperphosphatasia with impaired intellectual development syndrome 1 (Mabry syndrome)
**PTPN11**	Noonan Syndrome 1
**PURA**	Neurodevelopmental disorder with neonatal respiratory insufficiency, hypotonia, and feeding difficulties
**SETD1A**	Neurodevelopmental disorder with speech impairment and dysmorphic facies
**STXBP1**	Developmental and Epileptic Encephalopathy 4
**TRAPPC12**	Encephalopathy, progressive, early-onset, with brain atrophy and spasticity
**WDR81**	Hydrocephalus, congenital, 3, with brain anomalies
**WT1**	Denys-Drash Syndrome
**XYY, 8p23.3p23.2, 15q26.1q26.3**	XYY, 8p23.3p23.2 deletion, 15q26.1q26.3 duplication
**Post-intervention cohort**	
**12p13.33**	12p13.33 deletion
**15q11.2q13.1**	15q11.2q13.1 deletion, Prader-Willi Syndrome
**22q11.2**	22q11.2 deletion
**8p23.1**	8p23.1 deletion
**AGTPBP1**	Childhood-onset neurodegeneration with cerebellar atrophy
**ALDOB**	Hereditary fructose intolerance
**BRPF1**	Intellectual developmental disorder with dysmorphic facies and ptosis

**CA2**	Carbonic anhydrase II deficiency
**CHAMP1**	Neurodevelopmental disorder with hypotonia, impaired language, and dysmorphic features
**chr 21**	Trisomy 21
**CPS1**	Carbamoylphosphate synthetase 1 deficiency
**DBT**	Maple syrup urine disease type II
**DEPDC5**	Epilepsy, familial focal, with variable foci 1
**DMPK**	Myotonic dystrophy 1
**DNAH11**	Primary ciliary dyskinesia 7
**FOXF1**	Congenital alveolar capillary dysplasia with misalignment of pulmonary veins
**FOXG1**	Congenital variant of Rett syndrome
**FRAS1**	Fraser syndrome 1
**GLDC**	Glycine encephalopathy 1
**GLDC**	Glycine encephalopathy 1
**GPX4**	Sedaghatian type of spondylometaphyseal dysplasia
**IGHMBP2**	Spinal muscular atrophy with respiratory distress
**MOCS2**	Molybdenum cofactor deficiency type B
**MT-ATP6**	MT-ATP6-related disorder
**NDUFB3**	Mitochondrial complex I deficiency nuclear type 25
**NLRP3**	Neonatal onset multisystem inflammatory disease
**NOTCH2**	Alagille syndrome 2
**NSD1**	Sotos syndrome
**NSD1**	Sotos syndrome
**OCRL**	Lowe syndrome
**PIGN**	Multiple congenital anomalies-hypotonia-seizures syndrome 1
**POGZ**	White-Sutton Syndrome
**PRF1**	Familial hemophagocytic lymphohistiocytosis 2
**PLPBP**	Early-onset vitamin B6-dependent epilepsy 1
**RAC1**	Intellectual developmental disorder, autosomal dominant 48
**RAF1**	Noonan syndrome 5
**RBM10**	TARP Syndrome
**RRAS2**	Noonan syndrome 12
**SERAC1**	3-methylglutaconic aciduria with deafness, encephalopathy, and Leigh-like syndrome
**SKI**	Shprintzen-Goldberg craniosynostosis syndrome
**SMARCA4**	Coffin-Siris syndrome 4
**SOX9**	Campomelic dysplasia
**TGFBR2**	Loeys-Dietz syndrome 2
**TRMU**	Liver failure, transient infantile
**TWNK**	Mitochondrial DNA depletion syndrome 7 (hepatocerebral type)

**TYR**	Oculocutaneous albinism type IA
**VARS2**	Combined oxidative phosphorylation deficiency 20
**WT1**	Denys-Drash syndrome
**WT1**	Denys-Drash syndrome
**ZDHHC16**	ZDHHC16-related disorder
